# Accuracy of direct examination and culture as compared to the anatomopathological examination for the diagnosis of chromoblastomycosis: a systematic review^⋆^

**DOI:** 10.1016/j.abd.2021.09.007

**Published:** 2022-05-25

**Authors:** Jules Rimet Borges, Bárbara Álvares Salum Ximenes, Flávia Tandaya Grandi Miranda, Giordana Bruna Moreira Peres, Isabella Toscano Hayasaki, Luiz César de Camargo Ferro, Mayra Ianhez, Marco Tulio Antonio Garcia-Zapata

**Affiliations:** a Postgraduate Program in Health Sciences, Universidade Federal de Goiás, Goiânia, GO, Brazil; b Department of Tropical Medicine and Dermatology, Instituto de Patologia Tropical e Saúde Pública, Universidade Federal de Goiás, Goiânia, GO, Brazil; c Faculdade de Medicina, Universidade Federal de Goiás, Goiânia, GO, Brazil; d Department of Tropical Medicine and Dermatology Instituto de Patologia Tropical e Saúde Pública, Universidade Federal de Goiás, Goiânia, GO, Brazil

**Keywords:** Chromoblastomycosis, Diagnosis, Systematic review

## Abstract

**Background:**

Chromoblastomycosis is a skin infection caused by dematiaceous fungi that take the form of muriform cells in the tissue. It mainly manifests as verrucous plaques on the lower limbs of rural workers in tropical countries.

**Objectives:**

The primary objective of this review is to evaluate the accuracy of diagnostic methods for the identification of chromoblastomycosis, considering the histopathological examination as the reference test.

**Methods:**

MEDLINE, LILACS and Scielo databases were consulted using the terms “chromoblastomycosis” AND “diagnosis”. The eligibility criteria were: studies that evaluated the accuracy of tests for the diagnosis of chromoblastomycosis. Eleven studies were selected. Statistical analysis included the calculation of sensitivity and specificity of the diagnostic methods.

**Results:**

Considering the histopathological examination as the reference test, the culture showed a sensitivity (S) of 37.5% - 90.9% and a specificity (Sp) of 100%; while direct mycological examination showed S =  50% - 91.6% and Sp of 100% . Considering the culture as the reference test, the serology (precipitation techniques) showed S  of 36% - 99%; and Sp  of 80% - 100%; while the intradermal test showed S  of 83.3% - 100% and Sp  of 99.4% - 100%.

**Study limitations:**

The small number of studies and very discrepant sensitivity results among them do not allow the calculation of summary measures through a meta-analysis.

**Conclusions:**

Direct mycological examination, culture, intradermal test and serology show sensitivity and specificity values ​​for the diagnosis of chromoblastomycosis with no significant difference between the studies.

## Introduction

Chromoblastomycosis is a chronic skin and subcutaneous tissue infection acquired by inoculation of dematiaceous fungi of the *Herpotrichiellaceae* family through minor trauma. The most common species involved in the etiology of the disease are *Fonsecaea pedrosoi*, *Fonsecaea monophora* and *Cladophialophora carrionii*. Less commonly found agents are: *Phialophora verrucosa*, *Rhinocladiella aquaspersa*, *Exophiala* spp, in addition to other *Fonsecaea* species. It occurs predominantly in tropical and subtropical countries.^1^

The term chromoblastomycosis was first used in 1922 to define a skin disease caused by dematiaceous fungi that appear in the tissue as rounded brown structures called muriform cells. The disease has received different names in case reports, such as black blastomycosis, verrucous chromomycotic dermatitis, and verrucous blastomycotic dermatitis, Fonseca's disease, Pedroso's disease, and Carrion's disease. Since 1983, the term chromoblastomycosis has been used to differentiate this entity from infections caused by dematiaceous fungi that do not present as muriform cells in the host, called phaeohyphomycosis, which can be superficial, cutaneous, subcutaneous, and systemic. In the same year, it was established that the term chromomycosis should not be used because in some periods of history, it was used to define chromoblastomycosis and to avoid mistaking muriform cells for yeasts but, subsequently, it acquired a broader concept by some authors, including cases of phaeohyphomycosis.^1^, ^2^

The most affected population consists of male individuals who work with soil or plants, such as farmers, gardeners, and woodcutters, aged between 30 and 50 years, and most of the lesions appear in the lower limbs. Thus, the lack of protective gloves, shoes, and clothing during work is considered a risk factor. A possible genetic predisposition has been considered.^3^

In the environment, the fungus appears as hyphae, acquiring a muriform shape in the host, considered an element of resistance to the immune system defense mechanisms.^1^

The lesion appears as an erythematous macula or papule and develops into different clinical forms, depending on the cellular immune response: nodular, tumor, plaque, verrucous or cicatricial. It becomes pruritic and, in advanced cases, painful. The dissemination occurs by autoinoculation (facilitated by scratching), and by the lymphatic route. The disease can present complications: secondary bacterial infection, lymphedema, ankylosis, and the development of squamous cell carcinoma in chronic lesions.^4^

The diagnosis of chromoblastomycosis is confirmed by demonstrating the fungus presence on direct mycological examination, pathological examination, or culture.^1^

Direct examination with potassium hydroxide (KOH 10%‒40%) of scarification tissue from the lesion allows the identification of brown round or chestnut-shaped fungal elements, crossed by longitudinal and transverse septa, called muriform cells.^1^ Black dots on the lesion surface contain fungi that underwent transepidermal elimination and may guide the sample collection.^5^

Fungus growth in culture media occurs in four weeks, and the colony is filamentous and has a dark color. Culture on slides allows the differentiation between the genera *Fonsecaea*, *Phialophora*, *Cladophialophora* and *Rhinocladiella*.^1^

The histopathology of chromoblastomycosis is characterized by pseudoepitheliomatous hyperplasia, hyperkeratosis, and irregular acanthosis, alternating with areas of atrophy and abscesses; there is a granulomatous and suppurative inflammatory reaction with giant cells in the dermis. The fungi appear as thick-walled, dark brown ovoid or spherical structures, resembling “copper coins”, clearly visible, isolated or in clusters, inside giant cells, or free in the tissue.^6^

The diagnosis can also be attained by sequencing the fungus genetic material, a technique that allows identifying the species. Case series and reports have used polymerase chain reaction (PCR) and other molecular techniques to identify dematiaceous fungi in patients with chromoblastomycosis.^7^, ^8^, ^9^

Studies have been published evaluating the sensitivity and specificity of different serology techniques in patients with chromoblastomycosis caused by *F. pedrosoi* and *C. carrionii*, comparing them with healthy controls or patients with other fungal infections, such as sporotrichosis and paracoccidioidomycosis.^10^, ^11^

Intradermal tests obtained from cultures of *F. pedrosoi* and other agents have been used to compare results in patients with chromoblastomycosis and healthy controls or patients with other infections, but they are not commercially available.^12^, ^13^, ^14^

Recently, dermoscopic images of chromoblastomycosis lesions have been described and correlated with histopathological findings. Under dermoscopy, the authors found blackish-red spots, crusts, desquamation, ovoid yellow-orange structures on a whitish-pink background, and vascular polymorphism.^15^, ^16^, ^17^, ^18^

The aspect of the muriform cells on electron microscopy has already been described, characterizing the presence of melanossomes in the fungal cell – organelles that store melanin and their role in pathogenicity.^19^, ^20^, ^21^, ^22^ However, there are no studies evaluating the sensitivity or specificity of this method for the diagnosis of chromoblastomycosis, which is restricted to research centers.

To date, there has been no systematic review evaluating the accuracy of diagnostic tests in chromoblastomycosis. Moreover, individual studies evaluating the accuracy of diagnostic methods in chromoblastomycosis consider different tests as the reference test: culture, histopathological examination, and direct mycological examination. Finally, the role of the intradermal test and of serology, restricted to research settings, needs to be defined.

The aim of this review was to evaluate the accuracy of direct mycological examination and culture for the identification of chromoblastomycosis in patients with suspected disease, considering the histopathological examination as the reference test. Secondary objectives were the evaluation of the accuracy of the intradermal test and serology for the diagnosis of chromoblastomycosis considering the culture as the reference test, and also the accuracy of the histopathological examination in comparison to the direct mycological examination.

## Methods

The protocol registration number for this review in PROSPERO is CRD42020166336.

The Preferred Reporting Items for Systematic Reviews and Meta-Analyses (PRISMA) guideline, adapted for diagnostic accuracy studies, was used as a guide for the methodology of this review.^23^

Eligibility criteria were: case-control or cross-sectional/cohort studies evaluating the accuracy of complementary tests (intradermal test, serology, culture, direct mycological examination, anatomopathological examination) for the diagnosis of chromoblastomycosis in human patients, with varying degrees of severity, of all ages, of both sexes, of different ethnicities and from different geographic regions, considering as reference tests the anatomopathological examination, culture or direct examination. Animal studies were excluded, as well as case reports or series, reviews, guidelines, and studies with insufficient data to calculate the sensitivity and specificity of the described methods because for the statistical analysis to be performed, the studies should include true positive, false positive, true negative and false negative values. Therefore, they must disclose data on the results of the methods evaluated in patients with confirmed chromoblastomycosis and also in patients who received other diagnoses or who were considered healthy controls.

MEDLINE and LILACS databases were consulted by six independent researchers working in pairs, in January 2020, using the MESH terms “chromoblastomycosis” and “diagnosis”, with the Boolean operator AND; whereas the Scielo database was searched using the descriptor “chromoblastomycosis”. There were no restrictions on publication date or language. The platforms used in the search have an automatic blast effect and related terms such as “*cromoblastomicose*” and “chromoblastomycosis” are also included with this type of search. In the Scielo database, the addition of the term “diagnosis” did not result in the identification of any articles. The evaluation of titles and abstracts was performed by two independent researchers, and disagreements were resolved by a third reviewer. The reading of full texts to apply the eligibility criteria was also carried out by two independent researchers, and, at this stage, there was no divergence between the reviewers.

Data extraction was performed by two independent reviewers using a standardized clinical file. The study data (assessed index tests, reference test used, total number of patients in the study, and total number of patients included in the analyses) and inclusion criteria were extracted. Methodological criteria were also recorded: appropriate reference test, sample selection criteria (random, consecutive, or for convenience), patient follow-up (complete or with inappropriate exclusions), sample description (adequate or incomplete), study design (prospective or retrospective), blindness in the performance of the tests, the threshold of positivity used in the study and target condition definition (chromoblastomycosis) in the reference test. The numbers of true positive, true negative, false positive, and false negative values were also extracted to calculate the sensitivity and specificity of the evaluated tests. The evaluation of methodological quality, risk of bias, and applicability were analyzed using the QUADAS-2 tool, using a standardized form, by two independent reviewers. Four domains were evaluated: patient selection, performance and interpretation of the index test and the reference test, and patient flow (interval between tests, inclusion of the entire sample in the analyses, application of the same standard test to all participants).^24^

The statistical analysis was performed using the MADA package of the R application, which uses bivariate analysis with random effects. The studies were grouped according to the evaluated index test and the reference test used to diagnose the disease in the participants. The sensitivity and specificity of each method used in the study were calculated, as well as the confidence interval. The chi-square test was applied to analyze the homogeneity between the sensitivity and specificity values ​​of the same diagnostic technique used in different studies.

## Results

### Study selection

**Figure 1:**
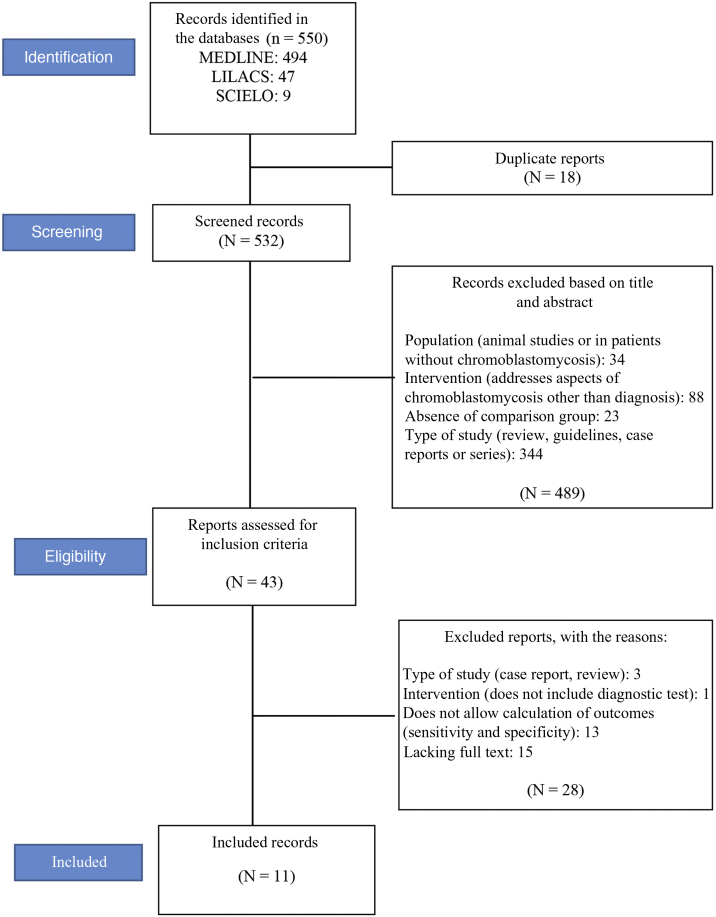
Flowchart of the selection of studies included in the systematic review.

A total of 550 records were located (see flowchart in Fig. 1): 494 in the MEDLINE database, 47 in LILACS, and nine in the Scielo database. Eighteen duplicate articles were removed, resulting in 532 articles whose titles and abstracts were screened. At this stage, 489 of the 532 studies were excluded. The eligibility criteria were applied to 43 studies, of which 11 met the eligibility criteria and were included in the review.^12^, ^13^, ^14^, ^25^, ^26^, ^27^, ^28^, ^29^, ^30^, ^31^, ^32^ Fig. 1 shows the study selection method flowchart.

In total, the 11 studies included 764 patients, 152 with chromoblastomycosis and 612 without the disease (healthy patients or patients with other mycoses such as sporotrichosis, paracoccidioidomycosis, or mycetoma).^12^, ^13^, ^14^, ^25^, ^26^, ^27^, ^28^, ^29^, ^30^, ^31^, ^32^ Two studies assessed the same sample of patients, testing different serological methods in each of them.^31^, ^32^ The studied patients were from India, China, Japan, Brazil, and Costa Rica. The participants characteristics are summarized in Table 1, and the methodological characteristics of the studies are shown in Table 2.

**Table 1:** Characteristics of the participants of the studies included in the review.

1st author, year of publication	Origin	Sample size (n)	Male: female ratio	Mean age in years
Bhat, 2016^25^	India (south)	25	16:9	49 (14‒74)
Bordoloi, 2015^14^	India (Assam)	46	NI	NI
Raj, 2015^26^	India (east)	20	NI	NI
Miranda, 2005^27^	Brazil (PA)	23	17:2^a^	56 (34‒75)^a^
Shao, 2020^28^	China	93	NI	NI
Iwatsu, 1979^12^	Japan	130	1:3^b^	52 (32‒73)
Iwatsu, 1982^13^	Japan	20	NI	NI
Buckley, 1966^30^	Brazil and Costa Rica	35	NI	NI
Marques, 2008^29^	Brazil (MA)	194	57:40	44 (17‒71)
Vidal, 2003^31^	Brazil (SP)	178^c^	NI	NI
Vidal, 2004^32^	Brazil (SP)	178^c^	NI	NI

Notes: MA, Maranhão; NI, Not informed; PA Pará; SP, São Paulo.

a There is no information about the sex and age of four patients who did not have a confirmed diagnosis of mycosis.

b Data were informed only for patients with chromoblastomycosis.

c Same sample.

**Table 2:** Characteristics of the studies included in the review.

1st author, year of publication	Period	Study design	Consecutive sample selection	Complete follow-up of patients	Reference test and positivity threshold	Proportion of patients submitted to the reference test (%)	Index test	Blindness in test performance
Bhat, 2016^25^	2005‒2013	Prospective	Consecutive	Yes	Histopathological ‒ Identification of muriform cells	100	Direct examination	NI
							Culture	
Bordoloi, 2015^14^	2013‒2014	Prospective	Consecutive	Yes	Histopathological and direct examination ‒ Identification of muriform cells	100	Culture	Yes
Raj, 2015^26^	24 months^a^	Prospective	Consecutive	No^d^	Histopathological and direct examination ‒ Identification of muriform cells	100	Culture	Yes
Miranda, 2005^27^	2000‒2004	Prospective	Consecutive	Yes^b^	Direct examination ‒ Identification of muriform cells	100	Direct examination with adhesive tape	No
							Histopathological	
							Culture	
Shao, 2020^28^	2010‒2018	Retrospective	Consecutive	No^e^	Histopathological ‒ Identification of muriform cells	100	Fluorescein-labeled chitinase	No
Iwatsu, 1979^12^	NI	Prospective	NI	Yes	Culture – growth of dematiaceous fungus	100	Intradermal test	Unclear
							Serology	
Iwatsu, 1982^13^	NI	Prospective	NI	Yes	Culture – growth of dematiaceous fungus	100	Intradermal test	Unclear
Buckley, 1966^30^	NI	Prospective	Convenience	Yes	Culture – growth of *F. pedrosoi*	100	Serology	No
Marques, 2008^29^	2002‒2003	Prospective	Consecutive	Yes	Histopathological, direct examination and culture - micromorphology	100^c^	Intradermal test	No
Vidal, 2003^31^	NI	Prospective	Convenience	Yes	Histopathological, direct examination – muriform cells; culture – growth of *F. pedrosoi*	100^c^	Serology	No
Vidal, 2004^32^	NI	Prospective	Convenience	Yes		100^c^	Serology	No

Notes: NI, Not informed.

a Unspecified period.

b All patients were submitted to the reference test (conventional microscopy) and the index test, which was the primary objective (direct microscopy with adhesive tape), but not all were submitted to culture and histopathological examination.

c 100% of patients with lesions (the study used healthy controls).

d Loss of follow-up of three patients.

e 54 of 93 patients completed the analyses.

### Risk of bias

**Figure 2:**
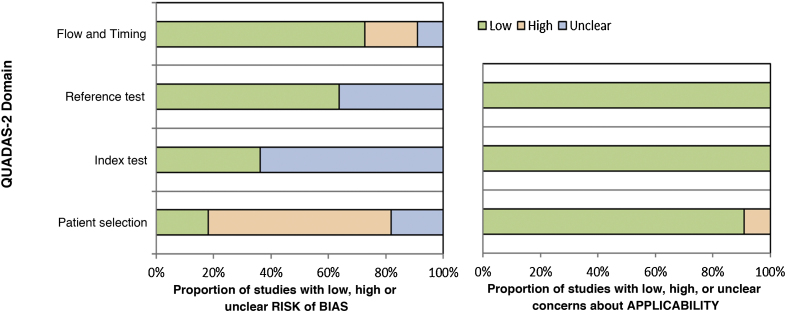
Proportion of studies with low, high or unclear risk of bias, according to the authors' judgment using the QUADAS-2 tool. Source: Prepared by the authors.

The results of the risk of bias assessment and concerns regarding the applicability of the studies are summarized in Table 3; the proportion of studies with low, high or unclear risk of bias is shown in Fig. 2.

**Table 3:** Result of the risk of bias assessment in the included studies, using the QUADAS-2 tool.

Study	Risk of bias				Concerns regarding applicability		
	Patient selection	Index test	Standard	Flow and timing	Patient selection	Index test	Standard
Bhat 2016	☺	☺	☺	☺	☺	☺	☺
Bordoloi 2015	☺	☺	☺	☺	☺	☺	☺
Raj 2015	☹	☺	☺	☹	☺	☺	☺
Miranda 2005	☺	☺	☺	☺	☺	☺	☺
Shao 2020	☹	?	?	☹	☹	☺	☺
Iwatsu 1979	?	?	☺	☺	☺	☺	☺
Iwatsu 1982	?	?	☺	☺	☺	☺	☺
Buckley 1966	☹	?	☺	☺	☺	☺	☺
Marques 2008	☹	?	?	?	☺	☺	☺
Vidal 2003	☹	?	?	☺	☺	☺	☺
Vidal 2004	☹	?	?	☺	☺	☺	☺

☺, Low risk; ☹, High risk;? , Unclear risk.

The articles by Bhat et al. (2016), Bordoloi et al. (2015), and Miranda, Silva (2005) showed a low risk of bias.^14^, ^25^, ^27^ The study by Raj et al. (2015) showed a risk of bias in patient selection and flow due to the loss of follow-up of three participants who were not included in the analysis.^26^ The risk of bias in the study by Shao et al. (2020) occurred because the sample used for the pathological examination included tissues other than skin and subcutaneous tissue, such as lung biopsy samples; in addition, there is no information about blindness of the tests.^28^ The studies by Iwatsu et al. (1979, 1982) show an unclear risk of bias because they do not report the sample selection criteria nor whether there was blindness in the performance of the tests.^12^, ^13^ In the study by Buckley, Murray (1966), the risk of bias in patient selection is high because the sample was selected for convenience and the risk of bias in the index test is unclear, as there is no information on whether the intradermal test was interpreted without knowledge of the reference test (culture).^30^ The study by Marques et al. (2008) included the selection of healthy controls, but there is no information about blindness in the performance of the tests, and there is no information on whether all the patients were submitted to the same reference test (it informs that direct mycological, anatomopathological examination and culture were performed, but there are no individual patient data).^29^ In the studies by Vidal (2003 and 2004), there was a group of healthy controls in the sample, and there was no information about blindness during test performance.^31^, ^32^

### Individual results

The evaluated outcomes (sensitivity and specificity of the tests for the diagnosis of chromoblastomycosis) are shown in Table 4.

**Table 4:** Individual results of studies included in the review.

1st author, year of publication	Reference test	Index test	TP	FP	TN	FN	S	Sp
Bhat, 2016^25^	AP	Direct examination	8	0	9	8	0.50	1.00
		Culture	6	0	9	10	0.375	1.00
Bordoloi, 2015^14^	AP and DME	Culture	5	0	40	1	0.83	1.00
Raj, 2015^26^	AP and DME	Culture	3	0	12	2	0.60	1.00
Miranda, 2005^27^	DME	VAT	11	0	11	1	0.916	1.00
		AP	6	0	6	0	1.00	1.00
		Culture	10	0	2	1	0.909	1.00
Shao, 2020^28^	AP	FLC	1	0	87	5	0.167	1.00
Iwatsu, 1979^12^	Culture	Intradermal test	8	0	42	0	1.00	1.00
		Serology	8	0	108	14	1.00	0.885
Iwatsu, 1982^13^	Culture	Intradermal test	5	0	14	1	0.833	1.00
Buckley, 1966^30^	Culture	Serology	12	0	22	1	0.923	1.00
Marques, 2008^29^	Culture, DME and AP	Intradermal test	18	1	173	2	0.90	0.994
Vidal, 2003^31^	Culture, DME and AP	Serology DID	32	28	112	6	0.53	0.96
		Serology CIE	41	19	106	12	0.68	0.905
		Serology ELISA	45	15	97	21	0.78	0.83
Vidal, 2004^32^	Culture, DME and AP	Serology IE	34	26	118	0	0.54	1.00
		Serology IB 54 kDa	58	2	118	0	0.967	1.00
		Serology IB 66 kDa	43	17	100	18	0.717	0.847

Notes: 54 kDa, Antigenic fraction (from *Fonsecaea pedrosoi*) of 54 kilodaltons; 66 kDa, Antigenic fraction (from *Fonsecaea pedrosoi*) of 66 kilodaltons; AP, Anatomopathological; CIE, Counterimmunoelectrophoresis; Sp, Specificity; ELISA, Enzyme-linked immunosorbent assay; DME, Direct Mycological Examination; FLC, Fluorescein-Labeled Chitinase; FN, False Negative; FP, False Positive; IB, Immunoblotting; DID, Double Immunodiffusion; IE, Immunoelectrophoresis; S, Sensitivity; VAT, Direct Examination with Vinyl Adhesive Tape; TN, True Negative; TP, True Positive.

### Summary of results

In the studies included in this review, culture sensitivity ranged from 37.5% to 90.9%; and specificity was 100%; sensitivity of direct mycological examination ranged from 50% to 91.6%; specificity was 100%; sensitivity of the intradermal test ranged from 83.3% to 100%; specificity ranged from 99.4% to 100%; sensitivity of serology ranged from 36% to 99%; and specificity from 80% to 100%; according to the technique used, the anatomopathological examination sensitivity ranged from 91% to 97%; and the specificity, from 92% to 100%.

Some diagnostic methods were assessed in a single publication: some serology techniques (enzyme immunoassay, immunoblotting, immunoelectrophoresis), fluorescein-labeled chitinase, and direct examination with adhesive tape, and their results are shown in Table 4. Studies that evaluated the same index test and the same reference test in the patients were pooled and analyzed together, as shown below.

**Figure 3:**
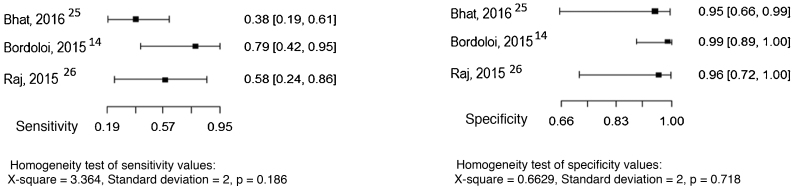
Sensitivity and specificity of the culture for the diagnosis of chromoblastomycosis, considering histopathological examination as the reference test. Source: Prepared by the authors, using the R application.

**Figure 4:**
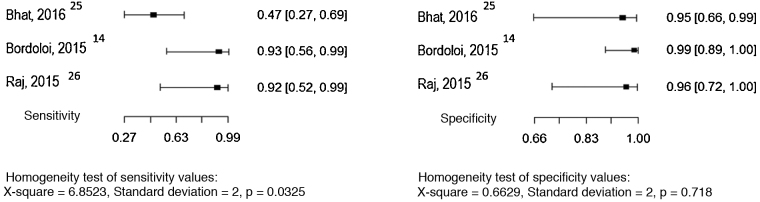
Sensitivity and specificity of direct mycological examination for the diagnosis of chromoblastomycosis, considering histopathological examination as the reference test. Source: Prepared by the authors, using the R application.

Three studies evaluated culture and the direct mycological examination as index tests, considering the histopathological examination as the reference test.^14^, ^25^, ^26^ The sensitivity and specificity values ​​found in the studies are shown in Figure 3, Figure 4.

**Figure 5:**
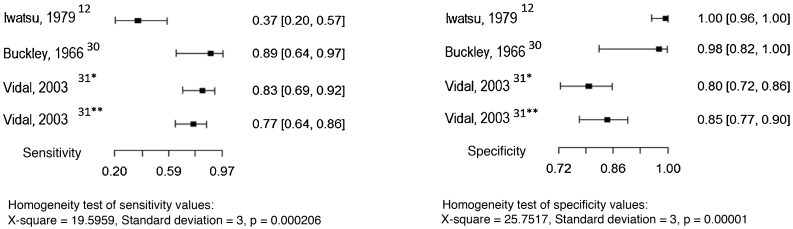
Sensitivity and specificity of serology (precipitation techniques) for the diagnosis of chromoblastomycosis, considering the culture as the reference test. Source: Prepared by the authors, using the application R. *Double Immunodiffusion; **Counterimmunoelectrophoresis.

Three studies evaluated serological precipitation techniques as the index test, considering culture as the reference test.^12^, ^30^, ^31^ In one of the studies, the patients were submitted to two precipitation techniques: double immunodiffusion and counterimmunoelectrophoresis.^31^ The sensitivity and specificity values ​​found in the studies are shown in Fig. 5.

**Figure 6:**
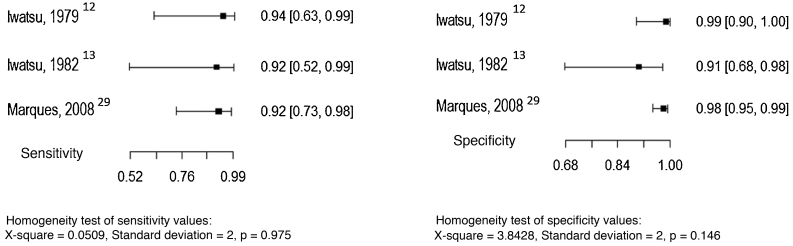
Sensitivity and specificity of the intradermal test for the diagnosis of chromoblastomycosis, considering the culture as the reference test. Source: Prepared by the authors, using the R application.

Three studies evaluated the intradermal examination as the index test, considering culture as the reference test.^12^, ^13^, ^29^ The sensitivity and specificity values ​​found in the studies are shown in Fig. 6.

**Figure 7:**
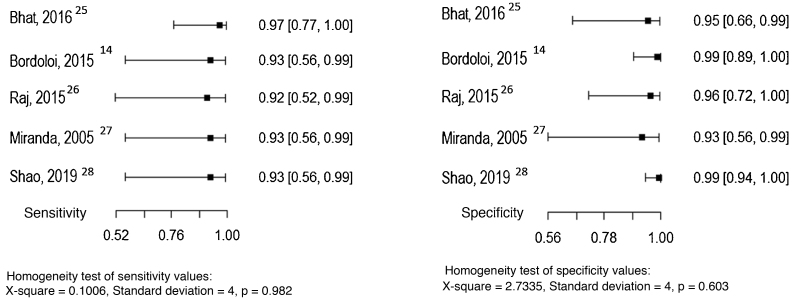
Sensitivity and specificity of the histopathological examination for the diagnosis of chromoblastomycosis, considering the direct mycological examination as the reference test. Source: Prepared by the authors, using the R application.

Five studies allowed the assessment of the histopathological examination as the index test, considering the direct mycological examination as the reference test.^14^, ^25^, ^26^, ^27^, ^28^ The sensitivity and specificity values ​​found in the studies are shown in Fig. 7.

Due to the small number of studies and the p-values ​​in the homogeneity tests, it is not possible to report an estimated pooled value of sensitivity and specificity.

## Discussion

The number of identified studies was small. For most of the assessed diagnostic techniques, there are only three published studies evaluating sensitivity and specificity. Moreover, in many of these studies, there was a high risk of bias in patient selection due to the choice of a convenience sample rather than a consecutive or random one. The sensitivity and specificity results found in publications that evaluated the same test were also very heterogeneous or divergent, and it was not possible to calculate a summary measure (pooled sensitivity and specificity) with statistical significance.

The sensitivity and specificity of serology for the diagnosis of chromoblastomycosis depend on the technique used. Precipitation techniques showed sensitivity and specificity levels above 80%, but these tests are not commercially available, and the technique lacks standardization. Additionally, studies that evaluated the diagnostic accuracy of serology used controls with a previously known diagnosis (healthy patients or patients with other mycoses), which represents a selection bias risk.^1^, ^12^, ^13^, ^30^, ^31^, ^32^

The intradermal test has sensitivity and specificity levels >90% but is species-specific and not available for use in daily practice.^3^, ^12^, ^29^

Culture sensitivity in the assessed studies ranged from 37.5% to 83%, with a specificity of 100%, and it is routinely used in clinical practice.^14^, ^25^, ^26^ Although molecular tests are necessary for species identification, the micromorphology study in culture on slides may suggest the genus and have an implication in the prognosis, increasing the importance of performing this test in clinical practice.^1^

Direct mycological examination, which is less frequently used in everyday life, has a sensitivity that ranges from 50% to 100%, and a specificity of 100% in the studies included in this review.^14^, ^26^, ^27^ In the study that compared different methods to obtain the sample, sample collection with adhesive tape showed a sensitivity of 91.6% and specificity of 100%, while the conventional technique showed a sensitivity and specificity of 100%.^27^

Some studies suggest collecting material for microscopy and culture from the black spots found in the lesion.^1^, ^4^ Dermoscopy facilitates the observation of these spots. Their absence in some forms of the disease, such as the cicatricial form, may explain the difference in sensitivity and specificity of this method between different evaluated populations, but further studies are needed to analyze this possibility.^15^, ^16^

Histopathological examination was used as the reference test in most of the selected studies. In the studies included in this review, the sensitivity of this method, when compared to the direct mycological examination, ranged from 92% to 97%, and the specificity from 93% to 99%.^14^, ^25^, ^26^, ^27^, ^28^ This method has been used in daily practice, as it allows the differential diagnosis with non-infectious diseases, such as lichen simplex chronicus and cutaneous lupus, as well as with other infections such as tuberculosis, cutaneous leishmaniasis, leprosy, histoplasmosis, paracoccidioidomycosis, and sporotrichosis. Moreover, in chronic cases, it allows the diagnosis of one of the most serious complications of the disease, which is the development of squamous cell carcinoma.^1^, ^25^, ^29^

In daily practice, the dermatologist uses anatomopathological examination and fungal culture of material obtained through biopsy of skin lesions for the diagnosis of chromoblastomycosis. The results of this review show that direct mycological examination, a widely available and low-cost option, has adequate sensitivity and specificity for routine use in suspected cases of chromoblastomycosis. The use of serology should be encouraged, as well as intradermal testing, but these tests are not commercially available; the sensitivity and specificity of these techniques vary according to the causal agent, as shown by the studies included in this review. Thus, they require prior knowledge of the most prevalent agents in each region, emphasizing the importance of the culture, which identifies the genus according to the morphology, or molecular techniques, such as polymerase chain reaction, which determine the species of fungus identified in the examined material.

The limitations of this review include: a small number of studies, lack of information about blindness in the performance of index tests, some studies with the sample selected for convenience, or in which the selection criteria were not established. Additionally, data on gender and age of participants are lacking in many studies, which limits the external validity of the results.

## Conclusion

Histopathological examination allows the identification of muriform cells, which are characteristic of the disease and is considered a reference test in most studies on chromoblastomycosis. Direct mycological examination, a low-cost technique, is a test with a sensitivity >50% and specificity >90% for the diagnosis of chromoblastomycosis in the evaluated studies, and its use in clinical routine is recommended after collecting samples from the black spots present on the lesion surface. Culture provides information about the morphology of the agent, with implications on the prognosis which should be evaluated in future studies. It is also suggested to perform research on the accuracy of molecular tests and the intradermal test in comparison to the histological examination for the diagnosis of chromoblastomycosis, as well as further research with serological tests performed with consecutive or random sample selection in patients with suspected disease, aiming to assess the applicability of these methods in daily practice.

## Financial support

None declared.

## Authors' contributions

Jules Rimet Borges: Critical review of the literature; collection, analysis and interpretation of data; statistical analysis; approval of the final version of the manuscript; design and planning of the study; drafting and editing of the manuscript; critical review of the manuscript.

Bárbara Álvares Salum Ximenes: Critical review of the literature; collection, analysis and interpretation of data.

Flávia Tandaya Grandi Miranda: Collection, analysis and interpretation of data; critical review of the literature.

Giordana Bruna Moreira Peres: Collection, analysis and interpretation of data; critical review of the literature.

Isabella Toscano Hayasaki: Collection, analysis and interpretation of data; critical review of the literature.

Luiz César de Camargo Ferro: Collection, analysis and interpretation of data; critical review of the literature.

Mayra Ianhez: Approval of the final version of the manuscript; design and planning of the study; effective participation in research orientation; critical review of the manuscript.

Marco Tulio Antonio Garcia-Zapata: Approval of the final version of the manuscript; design and planning of the study; effective participation in research orientation; critical review of the manuscript.

## Conflicts of interest

None declared.
